# Comparison of the protective effects of resveratrol and pterostilbene against intestinal damage and redox imbalance in weanling piglets

**DOI:** 10.1186/s40104-020-00460-3

**Published:** 2020-06-01

**Authors:** Hao Zhang, Yanan Chen, Yueping Chen, Shuli Ji, Peilu Jia, Yue Li, Tian Wang

**Affiliations:** 1 College of Animal Science and Technology, Nanjing Agricultural University, Nanjing, 210095 Jiangsu China; 2 Postdoctoral Research Station of Clinical Veterinary Medicine, College of Veterinary Medicine, Nanjing Agricultural University, Nanjing, 210095 Jiangsu China; 3 Shanghai Key Laboratory of Veterinary Biotechnology, Shanghai, 200240 China; 4 Postdoctoral Research Station of Food Science and Engineering, College of Food Science and Technology, Nanjing Agricultural University, Nanjing, 210095 Jiangsu China; 5 Institute of Animal Science, Jiangsu Academy of Agricultural Sciences, Nanjing, 210014 China

**Keywords:** Intestinal injury, Oxidative damage, Piglet, Pterostilbene, Resveratrol, Weaning stress

## Abstract

**Background:**

Evidence indicates that early weaning predisposes piglets to intestinal oxidative stress and increases the risk of intestinal dysfunction; however, there are minimal satisfactory treatment strategies for these conditions. This study investigated the potential of resveratrol and its analog, pterostilbene, as antioxidant protectants for regulating intestinal morphology, barrier function, and redox status among weanling piglets.

**Methods:**

A total of 144 piglets were selected at 21 days of age and randomly allocated into one of four treatment groups, each of which included six replicates. Piglets in a sow-reared control group were suckling normally between ages 21 and 28 days, while those in weaned groups were fed a basal diet, supplemented with either 300 mg/kg of resveratrol or with 300 mg/kg of pterostilbene. Parameters associated with intestinal injury and redox status were analyzed at the end of the feeding trial.

**Results:**

Early weaning disrupted the intestinal function of young piglets, with evidence of increased diamine oxidase activity and *D*-lactate content in the plasma, shorter villi, an imbalance between cell proliferation and apoptosis, an impaired antioxidant defense system, and severe oxidative damage in the jejunum relative to suckling piglets. Feeding piglets with a resveratrol-supplemented diet partially increased villus height (*P* = 0.056) and tended to diminish apoptotic cell numbers (*P* = 0.084) in the jejunum compared with those fed a basal diet. Similarly, these beneficial effects were observed in the pterostilbene-fed piglets. Pterostilbene improved the feed efficiency of weanling piglets between the ages of 21 and 28 days; it also resulted in diminished plasma diamine oxidase activity and *D*-lactate content relative to untreated weaned piglets (*P* < 0.05). Notably, pterostilbene restored jejunal antioxidant capacity, an effect that was nearly absent in the resveratrol-fed piglets. Pterostilbene reduced the malondialdehyde and 8-hydroxy-2´-deoxyguanosine contents of jejunal mucosa possibly through its regulatory role in facilitating the nuclear translocation of nuclear factor erythroid-2-related factor 2 and the expression levels of NAD(P)H quinone dehydrogenase 1 and superoxide dismutase 2 (*P* < 0.05).

**Conclusions:**

The results indicate that pterostilbene may be more effective than its parent compound in alleviating early weaning-induced intestinal damage and redox imbalance among young piglets.

## Background

Weaning is a critical event that can result in physiological, environmental, and social stress for piglets. This stress occurs particularly during the initial post-weaning period, which is frequently characterized by severe intestinal damage, infections, and diarrhea, compromising the ability of young piglets to resist subsequent diseases and resulting in severe economic losses in the pig industry [1, 2]. Oxidative stress, which results from an imbalance between the production of free radicals and the scavenging ability of the antioxidant defense system, has been implicated in the initiation and pathogenesis of intestinal disorders induced by early weaning [3–5]. The intestines are a primary site of pro-oxidant and antioxidant actions, and as such, the intestinal epithelium is vulnerable to damage associated with oxidative stress [6, 7]. The disruption of intestinal homeostasis by oxidative stress can impair mucosal barrier function and disturb the homeostasis of cell death and renewal, inducing several intestinal diseases in young animals [8]. This raises the possibility that the use of appropriate nutrients with favorable antioxidant activity could alleviate intestinal damage in early-weaned piglets.

Antioxidant compounds, specifically polyphenols from plants, are capable of scavenging free radicals and alleviating intestinal disorders associated with oxidative stress. In swine production, polyphenol compounds have exhibited potential in terms of promoting epithelial integrity, inhibiting pathogenic microorganisms, and improving the growth performance and feed efficiency of weanling piglets [9–12]. One such compound is resveratrol, which has attracted significant attention due to its diverse health-promoting properties [10, 13, 14]. It has been reported that resveratrol may have strong potential as an in-feed antibiotic alternative for promoting the growth performance, immune function, and microbial ecosystem of weanling piglets undergoing stressful situations [10]. Likewise, cellular studies have demonstrated that resveratrol is capable of mitigating intestinal barrier dysfunction and bacteria translocation caused by deoxynivalenol, a common mycotoxin contaminant that can trigger oxidative stress and impair intestinal homeostasis [15, 16].

However, several studies have raised concerns about the efficacy of *in vivo* resveratrol treatment due to its poor bioavailability (i.e., the limited amount of resveratrol that is able to exert beneficial effects in target tissues). Resveratrol undergoes extensive phase II metabolism in the intestine and the liver, such as glucuronidation and sulfation on the hydroxyl groups [17, 18]. These reactions result in the rapid clearance of resveratrol from the body, limiting its biological potency *in vivo* [17–20]. It is therefore important to identify any resveratrol derivative(s) that have superior bioavailability and favorable efficacy.

Pterostilbene is a natural compound primarily derived from blueberries and *Pterocarpus marsupium* heartwood [21]. As a dimethyl ether derivative of resveratrol, pterostilbene has similar antioxidant, anti-inflammatory, anti-obesity, and cholesterol-lowering properties as its parent compound [22–24]. Growing evidence has revealed that partial methylation causes pterostilbene to be more lipophilic, making it easier to be taken up by cells; therefore, pterostilbene exhibits higher intestinal absorption and utilization than resveratrol [18, 25–27]. Moreover, the 3,5-dimethoxy structure reduces the susceptibility of pterostilbene to conjugation metabolism, endowing it with better metabolic stability than its parent compound [18, 24]. In this regard, pterostilbene appears to be a promising candidate for alleviating early weaning-induced intestinal damage in young piglets. There is currently minimal information concerning the application of pterostilbene in swine production. This study was therefore conducted to evaluate the potential effects of resveratrol and pterostilbene on newly-weaned piglets’ growth performance, intestinal morphology, enterocyte proliferation and apoptosis, expression of critical tight junction complexes, and mucosal redox status.

## Materials and methods

### Animals and treatments

The protocols used in the animal experiments were approved by the Nanjing Agricultural University Institutional Animal Care and Use Committee (Permit number SYXK-2017–0027). A total of 144 male piglets (Duroc × Landrace × Yorkshire) with similar body weights were selected at 21 days of age. They were then divided into four treatment groups: a sow-reared control group (SR-CON), a weaned control group (W-CON), a weaned resveratrol group (W-RSV), and a weaned pterostilbene group (W-PT). Each group consisted of six replicates, each of which included six piglets. According to the experimental design of previous studies [4, 28], piglets in the SR-CON group continued to be nursed by sows from 21 to 28 days of age; the remaining groups were moved from the farrowing room to their assigned pen. Between 21 and 28 days of age, they were fed a basal diet supplemented with: 300 mg/kg of resveratrol (W-RSV), 300 mg/kg of pterostilbene (W-PT), or no supplementation (W-CON). The dosages of resveratrol and pterostilbene added to the diet of weaned piglets were selected based on an independent pre-study, as well as the results reported by our colleagues and other groups [29, 30]. A greater degree of feed efficiency was observed for weaned piglets between the ages of 21 and 42 days when pterostilbene was provided at 250 or 500 mg/kg of diet (unpublished). Resveratrol has been proven to improve the jejunal antioxidant ability of weaned piglets at 42 days of age and prevent the muscular lipid peroxidation of finishing pigs at slaughter [29, 30]. Therefore, the same dose, 300 mg/kg, was selected for both resveratrol and pterostilbene in this study. The basal diet was formulated to meet the nutritional requirements for piglets (see Table 1), according to the National Research Council (2012) guidelines. Temperatures of between 29 °C and 31 °C were maintained in the farrowing and nursery pens. Feed and water were freely available. All piglets were weighed at 21 and 28 days of age to calculate their average daily gain (ADG), while the feed intake for piglets in each pen of the W-CON, W-RSV, and W-PT groups was registered to calculate their average daily feed intake (ADFI) and feed efficiency (FE).

**Table 1:** Composition and nutrient levels of the diets (%, as-fed basis unless otherwise stated)

Items	Contents
Maize	62.78
Soybean meal	15.00
Fermented soybean meal	7.00
Extruded soybean	7.00
Soy protein isolate	1.30
Soyabean oil	2.00
CaHPO_4_	1.80
Limestone	0.80
Salt	0.35
*L*-lysine-HCl, 78%	0.52
*L*-methionine	0.13
*L*-threonine	0.15
*L*-isoleucine	0.10
*L*-tryptophan	0.01
*L*-histidine	0.01
Calcium propionate, 50%	0.05
Premix^a^	1.00
Total	100.00
Nutrient levels^b^	
Digestible energy, Mcal/kg	3.47
Metabolizable energy, Mcal/kg	3.30
Crude protein	20.36
Total lysine	1.51
Total methionine	0.46
Total methionine + cystine	0.86
Total threonine	0.94
Total tryptophan	0.40
Total histidine	0.77
Total isoleucine	0.79
Total valine	1.20
Total calcium	0.82
Total phosphorus	0.65

^a^Provide the following per kg complete diet: Vitamin A, 8,000 IU; Vitamin D_3_, 3,000 IU; Vitamin E, 20 IU; Vitamin K_3_, 3 mg; Vitamin B_1_, 2 mg; Vitamin B_2_, 5 mg; Vitamin B_6_, 7 mg; Vitamin B_12_, 0.02 mg; Niacin, 30 mg; Pantothenic acid, 15 mg; Folic acid, 0.3 mg; Biotin, 0.08 mg; Choline chloride, 500 mg; Fe (from ferrous sulfate), 110 mg; Cu (from copper sulfate), 7 mg; Mn (from manganese sulfate), 5 mg; Zn (from zinc sulfate), 110 mg; I (from calcium iodate), 0.3 mg; Se (from sodium selenite), 0.3 mg

^b^All nutrient levels were analyzed values, except digestible energy and metabolizable energy

### Sample collection

At 28 days of age, six piglets (one from each replicate) were randomly selected from each group for sampling. Ten milliliters of blood were drawn from the anterior vena cava, collected in chilled heparinized vacutainers, and centrifuged at 4,000 × *g* for 15 min at 4 °C to obtain plasma samples, which were stored at − 80 °C until analysis. The piglets were then euthanized by electrical stunning and exsanguination. The entire intestine was immediately removed and placed on a cold tray to collect the jejunum. Two continuous segments were carefully cut from the middle of the whole jejunum for histological assay and mucosa collection. Sections of approximately 1.5 cm in length were fixed in fresh, chilled 4% paraformaldehyde for morphometric evaluation and histochemical staining. Portions of the jejunum measuring approximately 20 cm were cut longitudinally and cleaned with ice-cold phosphate buffer solution (PBS). Mucosa samples were collected using scraping by sterile glass microscope slides; they were then snap-frozen in liquid nitrogen and stored at − 80 °C for subsequent analysis.

### Determination of diamine oxidase (DAO) activity and *D*-lactate content

Plasma DAO activity was measured using a spectrophotometer, as described previously [31]. The kit (#A088–1-1) was obtained from the Nanjing Jiancheng Institute of Bioengineering (Nanjing, Jiangsu, China). The concentration of *D*-lactate in the plasma samples was determined using a commercial kit (#AAT-13811) purchased from AAT Bioquest (Sunnyvale, CA, USA). All procedures were performed with strict adherence to the manufacturers’ guidelines.

### Jejunal morphological examination

After being fixed in paraformaldehyde solution at room temperature for 24 h, the jejunal tissue specimens were dehydrated using an upgraded series of ethanol and xylene and then processed into paraffin blocks. A cross section with a thickness of 5 μm was cut from each specimen and stained with hematoxylin and eosin. Twenty well-oriented and intact villi and adjacent crypts were randomly selected for the measurements of villus height (VH) and crypt depth (CD) per slide by an assessor blinded to the treatments using optical microscopy (Nikon Eclipse 80i; Nikon, Tokyo, Japan) and NIS-Elements 3.0 Imaging Software.

### Determination of jejunal antioxidant enzyme activities and metabolite contents

Approximately 300 mg of frozen jejunal mucosa was immediately placed in a 1:9 (wt/vol) 0.86% saline solution and homogenized using an Ultra-Turrax homogenizer (Tekmar Co., Cincinnati, OH, USA). Tissue sediment was then removed by centrifugation at 4000 × *g* for 20 min at 4 °C, and the supernatant was obtained for analysis. Based on manufacturers’ instructions, commercial kits were used to determine superoxide dismutase (SOD; #A001–1-2; Nanjing Jiancheng Institute of Bioengineering), glutathione peroxidase (GSH-Px; #A005–1-2; Nanjing Jiancheng Institute of Bioengineering), catalase (CAT; #A007–1-1; Nanjing Jiancheng Institute of Bioengineering), and glutathione S-transferase (GST; #GST-2-W; Suzhou Comin Biotechnology Co., Ltd., Suzhou, Jiangsu, China) activities, as well as reduced glutathione (GSH; #A006–1-1; Nanjing Jiancheng Institute of Bioengineering) and malondialdehyde (MDA; #A003–1-2; Nanjing Jiancheng Institute of Bioengineering) contents. The total protein concentration of each homogenate sample was measured using the Enhanced Bicinchoninic Acid Protein Assay Kit (#P0010), according to the manufacturer’s instructions, with bovine serum albumin serving as the standard (Beyotime Institute of Biotechnology, Nantong, Jiangsu, China).

### Analysis of the extent of jejunal DNA oxidative damage

The DNA from jejunal mucosa was extracted using the TIANamp Genomic DNA kit (#DP304–03; Tiangen, Beijing, China). Following quantification using a NanoDrop ND-1000UV spectrophotometer (NanoDrop Technologies, Wilmington, DE, USA), the DNA samples were tested using a commercial kit purchased from Cayman Chemical (Ann Arbor, MI, USA) to determine the concentration of 8-hydroxy-2´-deoxyguanosine (8-OHdG; #589320-96S). For inter-sample comparison, the results were normalized against DNA concentrations for each sample.

### Immunohistochemistry

Thin sections (5 μm) from the jejunum were taken using a one-step terminal deoxynucleotidyl transferase dUTP nick end labeling (TUNEL) kit (#A113–03; Vazyme Biotech Co., Ltd., Nanjing, Jiangsu, China) to determine the apoptotic rate [32]. Each tissue section was permeabilized with a proteinase K solution (20 μg/mL) at room temperature for 20 min. After washing twice with phosphate buffer solution, the TUNEL-mixed reagents were added to the sections and incubated in a dark location at 37 °C for 60 min. We used 4′-6-diamidino-2-phenylindole (DAPI; #E607303; Beyotime Institute of Biotechnology) to label the nuclei. For each section, a blind observer used a fluorescence microscope (Nikon Eclipse C1; Nikon, Tokyo, Japan) to count the TUNEL-positive cells (red cells) in 15 random fields. The apoptotic rate was calculated as the number of apoptotic cells on all epithelial cells of the villi, based on the method employed by Słupecka et al. [33].

Ki-67 is a specific and reliable marker for the proliferation of cells [34]. The slides were deparaffinized and placed in an antigen-retrieval target solution (#G1203; Wuhan Servicebio Technology Co., Ltd., Wuhan, Hubei, China); they were then microwaved for 25 min to remove endogenous peroxidase and block nonspecific bindings. The sections were incubated with a polyclonal rabbit anti-pig antibody against Ki-67 (#ab15580; Abcam, Cambridge, MA, USA; 1:750) for 60 min and a CoraLite594-conjugated goat anti-rabbit secondary antibody (#SA00013–4; Proteintech, Chicago, IL, USA) for 30 min. The nuclei were then stained by DAPI, as described above. In each slice, 15 random crypts were measured by an assessor blinded regarding the source of the samples. The cell proliferation index was calculated by dividing the number of Ki67-positive cells by the total number of crypt cells, based on the methods of previous studies [32, 35].

### Total RNA isolation and RT-qPCR analysis

The extraction of total RNA and its reverse transcription were performed using the methods delineated in our previous study [36]. After retrieving the snap-frozen jejunal mucosal samples, we isolated their total RNA using TRIzol Reagent (#9109), as suggested in the manufacturer’s manual (TaKaRa Biotechnology, Dalian, Liaoning, China). Extracted RNA was dissolved in 50 μL of ultra-pure water. We used a NanoDrop ND-1000UV spectrophotometer (NanoDrop Technologies) to measure the purity and concentration of total RNA at 260 and 280 nm. Electrophoresis on a 1.5% agarose gel that had been stained Ultra GelRed™ (#GR501–01; Vazyme Biotech Co., Ltd.) was used to verify RNA integrity.

One microgram of total RNA was then reverse-transcribed into complementary DNA using the PrimeScript™ RT Reagent Kit (#RR036A; TaKaRa Biotechnology). A real-time polymerase chain reaction (PCR) was performed on a QuantStudio 5 Real-Time PCR System (Applied Biosystems, Life Technologies, CA, USA) using the ChamQTM SYBR® qPCR Master Mix Kit (#Q311–02; Vazyme Biotech Co., Ltd.), as recommended in the manufacturer’s guidelines. The PCR process involved a 30-s pre-run at 95 °C, 40 cycles of denaturation at 95 °C for 5 s, and a 60 °C annealing step for 30 s. For the melting curve conditions, one denaturation cycle was performed at 95 °C for 10 s; the temperature was then increased from 65 to 95 °C at a rate of 0.5 °C/s. Table 2 includes details of the primer sequences for the following target and reference genes used in this study: NAD(P) H quinone dehydrogenase 1 (*NQO1*), heme oxygenase 1 (*HO1*), superoxide dismutase 1 (*SOD1*), superoxide dismutase 2 (*SOD2*), glutathione peroxidase 1 (*GPX1*), glutathione peroxidase 2 (*GPX2*), glutathione peroxidase 3 (*GPX3*), glutathione peroxidase 4 (*GPX4*), glutathione S-transferase alpha 1 (*GSTA1*), glyceraldehyde-3-phosphate dehydrogenase (*GAPDH*), and beta actin (*ACTB*). The relative expression abundance of each target gene was calculated in accordance with the 2^−ΔΔCt^ method, as elucidated previously [37].

**Table 2:** Primer sequences used for real-time PCR assay

Name^a^	Genbank^b^	Sequence (5′ → 3′)^c^	Length, bp
*NQO1*	NM_001159613.1	CATGGCGGTCAGAAAAGCAC	135
		ATGGCATACAGGTCCGACAC	
*HO1*	NM_001004027.1	TGATGGCGTCCTTGTACCAC	71
		GACCGGGTTCTCCTTGTTGT	
*SOD1*	NM_001190422.1	AAGGCCGTGTGTGTGCTGAA	118
		GATCACCTTCAGCCAGTCCTTT	
*SOD2*	NM_214127.2	GGCCTACGTGAACAACCTGA	126
		TGATTGATGTGGCCTCCACC	
*GPX1*	NM_214201.1	CCTCAAGTACGTCCGACCAG	85
		GTGAGCATTTGCGCCATTCA	
*GPX2*	NM_001115136.1	CTGGACGGGGAGAAGGTAGA	107
		TTGAGTTGGGTGAAGTCCCG	
*GPX3*	NM_001115155.1	GTATGGAGCCCTCACCATCG	122
		TCAGTTCAACGTACTGGCCC	
*GPX4*	NM_214407.1	TGTGTGAATGGGGACGATGC	135
		CTTCACCACACAGCCGTTCT	
*GSTA1*	NM_214389.2	ACACCCAGGACCAATCTTCTG	199
		AGTCTCAGGTACATTCCGGG	
*GAPDH*	NM_001206359.1	CCAAGGAGTAAGAGCCCCTG	125
		AAGTCAGGAGATGCTCGGTG	
*ACTB*	XM_003124280.5	TGGAACGGTGAAGGTGACAG	176
		CTTTTGGGAAGGCAGGGACT	

^a^*ACTB* Beta actin, *GAPDH* Glyceraldehyde-3-phosphate dehydrogenase, *GPX1* Glutathione peroxidase 1, *GPX2* Glutathione peroxidase 2, *GPX3* Glutathione peroxidase 3, *GPX4* Glutathione peroxidase 4, *GSTA1* Glutathione S-transferase alpha 1, *HO1* Heme oxygenase 1, *NQO1* NAD(P)H quinone dehydrogenase 1, *SOD1* Superoxide dismutase 1, *SOD2* Superoxide dismutase 2

^b^GenBank Accession Number

^c^Shown as the forward primer followed by the reverse primer

### Western blot analysis

Approximately 100 mg of jejunal mucosa was cut into pieces, mixed with lysis/radio immunoprecipitation assay buffer (#P0013B; Beyotime Institute of Biotechnology) to a final concentration of 10% (wt/vol), and then homogenized using a glass-glass homogenizer on ice. The homogenized tissue was then centrifuged at 12,000 × *g* for 5 min at 4 °C to obtain the supernatants. The nuclear protein of jejunal mucosa was isolated using a commercial kit (#P0028) obtained from Beyotime Institute of Biotechnology. The protein content in the supernatants was quantified using the Enhanced Bicinchoninic Acid Protein Assay Kit (#P0010; Beyotime Institute of Biotechnology). Equal amounts of protein were loaded and separated through 10% sodium dodecyl sulfate-polyacrylamide gel electrophoresis prior to being transferred onto polyvinylidene difluoride membranes (#IPVH00010; Millipore, Bedford, MA, USA).

After blocking with Tris-buffered saline combined with 0.2% Tween-20 (TBST) containing 5% (wt/vol) skimmed milk powder at room temperature for 1 h, the membranes were incubated overnight at 4 °C with the QuickBlock™ Primary Antibody Dilution Buffer (#P0256; Beyotime Institute of Biotechnology) containing the following appropriate primary antibodies: anti-nuclear factor erythroid 2-related factor 2 (NRF2; #ab92946; Abcam; 1:1,000 dilution), anti-histone H3 (#5192S; CST, Danvers, MA, USA; 1:1,000 dilution), anti-Kelch ECH-associating protein 1 (KEAP1; #ab196346; Abcam; 1:2,000 dilution), anti-HO1 (#ab13248; Abcam; 1:1,000 dilution), anti-NQO1 (#11451–1-AP; Proteintech; 1:1,000 dilution), anti-SOD1 (#10269–1-AP; Proteintech; 1:1,000 dilution), anti-SOD2 (#66474–1-Ig; Proteintech; 1:5,000 dilution), anti-occludin (OCLN; #13409–1-AP; Proteintech; 1:1,000 dilution), anti-tight junction protein 1 (ZO1; #21773–1-AP; Proteintech; 1:2,000 dilution), and anti-ACTB (#4970S; CST; 1:1,000 dilution).

The blots were then washed three times and incubated with the QuickBlock™ Secondary Antibody Dilution Buffer (#P0258; Beyotime Institute of Biotechnology), including corresponding secondary antibodies at room temperature for 90 min. They were subsequently washed three more times for 5 min each. The immunoreactive bands were then visualized using an enhanced chemiluminescence reagent (#WBKLS0100; Millipore, Bedford, MA, USA), captured with a Tanon 5200 Multi-Imaging System (Tanon Science & Technology Inc., Shanghai, China), and quantified using the Gel-Pro Analyzer program (Media Cybernetics, Silver Spring, MD, USA).

### Statistical analysis

The data were analyzed for the homogeneity of variances and normality using Levene’s and Shapiro-Wilk’s tests, respectively. Based on Li et al.’s methodology [32], the normal data were assessed for statistical significance using a one-way analysis of variance (ANOVA) and Tukey’s *post-hoc* test for pairwise comparisons. Conversely, the heterogeneous or non-normally-distributed data were analyzed using a non-parametric Kruskal-Wallis test, and pairwise differences in rank sums were evaluated using selected comparisons tests. All analyses were performed using the Statistical Package for the Social Sciences (SPSS) software (ver. 22.0 for Windows, SPSS Inc., Chicago, USA). A *P*-value of less than 0.05 was considered statistically significant, and a *P*-value of between 0.05 and 0.10 was regarded as a trend. Results are presented as mean and standard error (SE).

## Results

### Growth performance

Compared with the SR-CON group, piglets in each weaned group exhibited a significant decrease (*P* < 0.05) in ADG during the first week after weaning (Table 3). Neither resveratrol nor pterostilbene altered the ADG of weaned piglets compared with the W-CON piglets (*P* > 0.10). Pterostilbene produced an increased (*P* < 0.05) FE in weaned piglets between 21 and 28 days of age relative to the W-CON group, an effect that was absent in the W-RSV group (*P* > 0.10). In addition, no discernible alteration of ADFI was observed between the weaned groups (*P* > 0.10).

**Table 3:** Effects of resveratrol and pterostilbene on growth performance of piglets from 21 to 28 days of age^a^

Items^b^	SR-CON (Group I)	W-CON (Group II)	W-RSV (Group III)	W-PT (Group IV)	Contrast					
					I vs. II	I vs. III	I vs. IV	II vs. III	II vs. IV	III vs. IV
ADG^c^, g/d	271.03 ± 13.77	140.08 ± 10.03	155.16 ± 9.92	168.65 ± 5.91	< 0.001	< 0.001	< 0.001	0.731	0.235	0.791
ADFI^c^, g/d	NA	205.16 ± 19.04	213.49 ± 10.04	223.02 ± 12.47	NA	NA	NA	0.912	0.661	0.887
FE^c^, g/g	NA	0.69 ± 0.02	0.72 ± 0.02	0.76 ± 0.02	NA	NA	NA	0.377	0.033	0.348

^a^Values are means ± standard error, *n* = 6

^b^*ADG* Average daily gain, *ADFI* Average daily feed intake, *FE* Feed efficiency, *NA* Not available, *SR-CON* Piglets continued to be nursed by sows aged between 21 and 28 days, *W-CON* Piglets were fed a basal diet aged between 21 and 28 days, *W-RSV* Piglets were fed a diet supplemented with 300 mg/kg of resveratrol aged between 21 and 28 days, *W-PT* Piglets were fed a diet supplemented with 300 mg/kg of pterostilbene aged between 21 and 28 days

^c^One-way ANOVA test

### Plasma DAO activity and *D*-lactate content

**Fig. 1:**
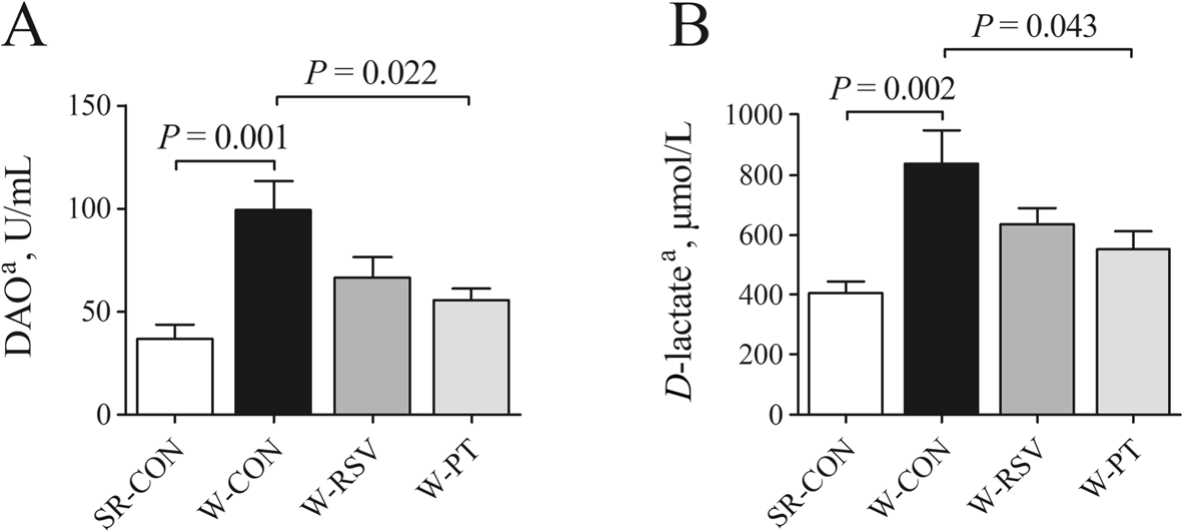
Effects of resveratrol and pterostilbene on the DAO (**a**) activity and *D*-lactate (**b**) content in the plasma of piglets. The column and its bar represent the mean value and standard error (*n* = 6 piglets/group), respectively. ^a^One-way ANOVA test. *DAO* diamine oxidase, *SR-CON* piglets continued to be nursed by sows aged between 21 and 28 days, *W-CON* piglets were fed a basal diet aged between 21 and 28 days, *W-RSV* piglets were fed a diet supplemented with 300 mg/kg of resveratrol aged between 21 and 28 days, *W-PT* piglets were fed a diet supplemented with 300 mg/kg of pterostilbene aged between 21 and 28 days

Compared with the SR-CON piglets, plasma DAO (Fig. 1a) activity and *D*-lactate (Fig. 1b) concentration increased (*P* < 0.05) by 171.3% and 107.0% in the W-CON piglets, respectively. Relative to the weaned piglets fed a basal diet, both DAO activity and *D*-lactate content significantly decreased (*P* < 0.05) for piglets fed a pterostilbene-supplemented diet; this reducing effect nearly restored the levels of these parameters to sow-reared amounts. However, resveratrol had no clear effect on circulating DAO activity or *D*-lactate content in the W-RSV piglets relative to the W-CON piglets (*P* > 0.10).

### Jejunal morphological analysis

**Fig. 2:**
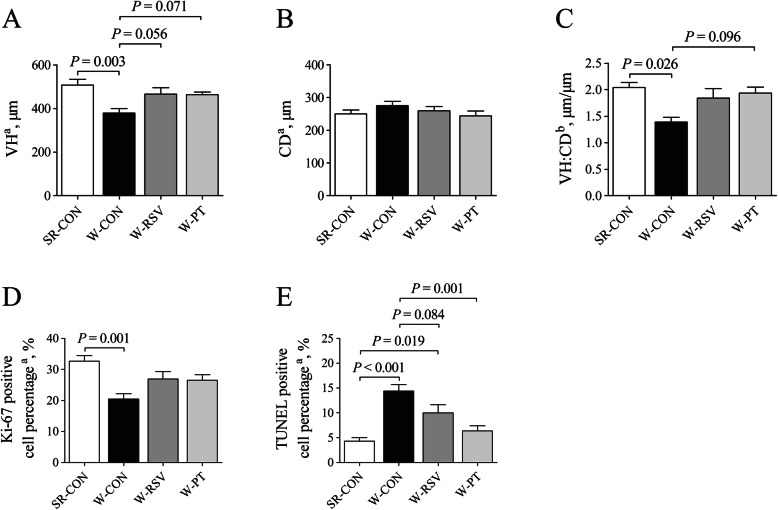
Effects of resveratrol and pterostilbene on VH (**a**), CD (**b**), VH:CD ratio (**c**), Ki-67 positive cell percentage (**d**), and TUNEL positive cell percentage (**e**) in the jejunum of piglets. The column and its bar represent the mean value and standard error (*n* = 6 piglets/group), respectively. ^a^One-way ANOVA test. ^b^Non-parametric Kruskal-Wallis test. *CD* crypt depth, *SR-CON* piglets continued to be nursed by sows aged between 21 and 28 days, *TUNEL* terminal deoxynucleotidyl transferase dUTP nick end labeling, *VH* villus height, *VH:CD* the ratio of villus height to crypt depth, *W-CON* piglets were fed a basal diet aged between 21 and 28 days, *W-RSV* piglets were fed a diet supplemented with 300 mg/kg of resveratrol aged between 21 and 28 days, *W-PT* piglets were fed a diet supplemented with 300 mg/kg of pterostilbene aged between 21 and 28 days

Early weaning resulted in marked reductions (*P* < 0.05) in jejunal VH (Fig. 2a) and the ratio of VH to CD (Fig. 2c) in the W-CON group relative to the SR-CON group. Resveratrol (*P* = 0.056) and pterostilbene (*P* = 0.071) tended to increase the jejunal VH of weaned piglets compared with the basal diet. In addition, the ratio of VH to CD exhibited an increased trend in the jejunum of piglets fed a pterostilbene-supplemented diet compared with those fed a basal diet with no supplement (*P* = 0.096). However, neither resveratrol nor pterostilbene altered the CD (Fig. 2b) of the jejunum of weaned piglets (*P* > 0.10).

**Fig. 3:**
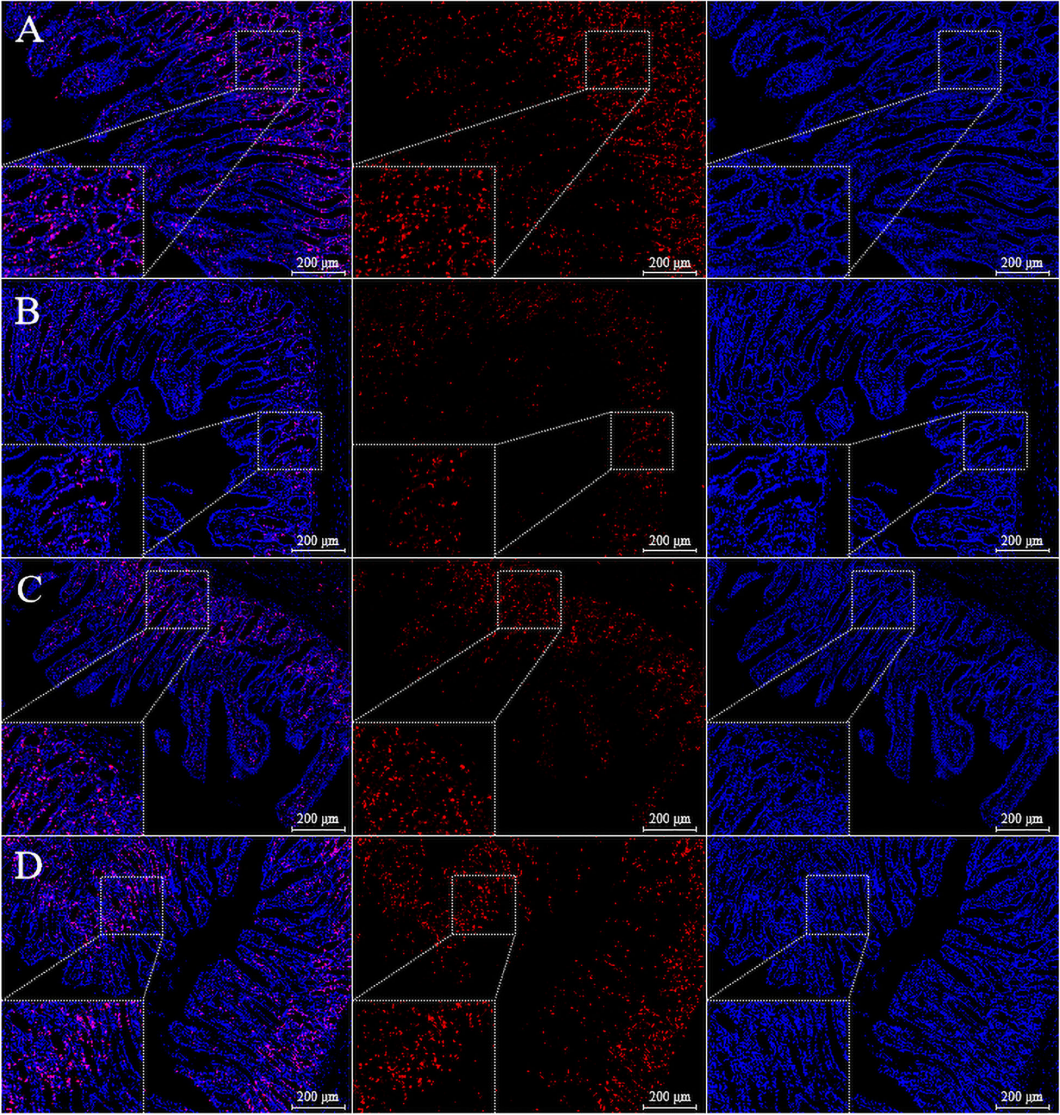
Ki-67 staining on immunofluorescence images in paraformaldehyde-fixed cross-sections from the jejunum of SR-CON (**a**), W-CON (**b**), W-RSV (**c**), and W-PT (**d**) groups (100 × and 400 × magnification). *SR-CON* piglets continued to be nursed by sows aged between 21 and 28 days, *W-CON* piglets were fed a basal diet aged between 21 and 28 days, *W-RSV* piglets were fed a diet supplemented with 300 mg/kg of resveratrol aged between 21 and 28 days, *W-PT* piglets were fed a diet supplemented with 300 mg/kg of pterostilbene aged between 21 and 28 days

**Fig. 4:**
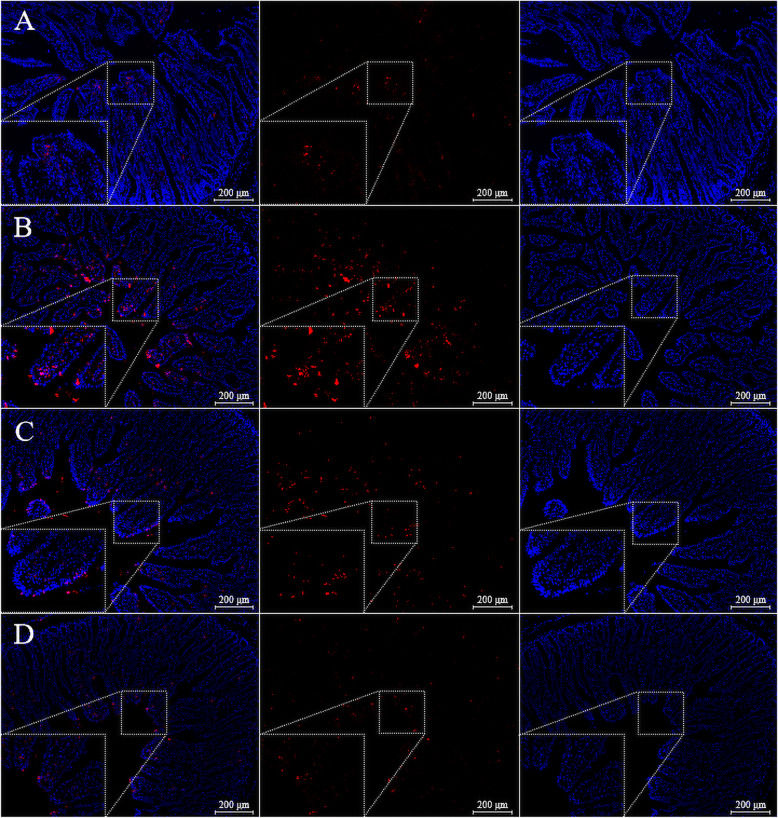
TUNEL staining on immunofluorescence images in paraformaldehyde-fixed cross-sections from the jejunum of SR-CON (**a**), W-CON (**b**), W-RSV (**c**), and W-PT (**d**) groups (100 × and 400 × magnification). *SR-CON* piglets continued to be nursed by sows aged between 21 and 28 days, *TUNEL* terminal deoxynucleotidyl transferase dUTP nick end labeling, *W-CON* piglets were fed a basal diet aged between 21 and 28 days, *W-RSV* piglets were fed a diet supplemented with 300 mg/kg of resveratrol aged between 21 and 28 days, *W-PT* piglets were fed a diet supplemented with 300 mg/kg of pterostilbene aged between 21 and 28 days

### Cell proliferation and apoptosis

Early weaning significantly diminished (*P* < 0.05) the numbers of Ki-67 positive cells (Fig. 2d; Fig. 3) in the jejunal crypt of untreated weanling piglets relative to piglets nursing the sow. However, this parameter failed to indicate any significant variation between the weaned groups (*P* > 0.10). Compared with the SR-CON piglets, the percentages of apoptotic cells (Fig. 2e; Fig. 4) in the jejunal villus of the W-CON and W-RSV groups were 235.0% and 132.0% greater (*P* < 0.05), respectively. Pterostilbene administration reduced (*P* < 0.05) the numbers of jejunal apoptotic cells by 55.8% in the W-PT group relative to the W-CON group. In addition, piglets fed a resveratrol-diet exhibited a tendency to decrease (*P* = 0.084) in jejunal apoptotic cell percentage relative to their counterparts that were fed a basal diet.

### Jejunal OCLN and ZO1 expression

**Fig. 5:**
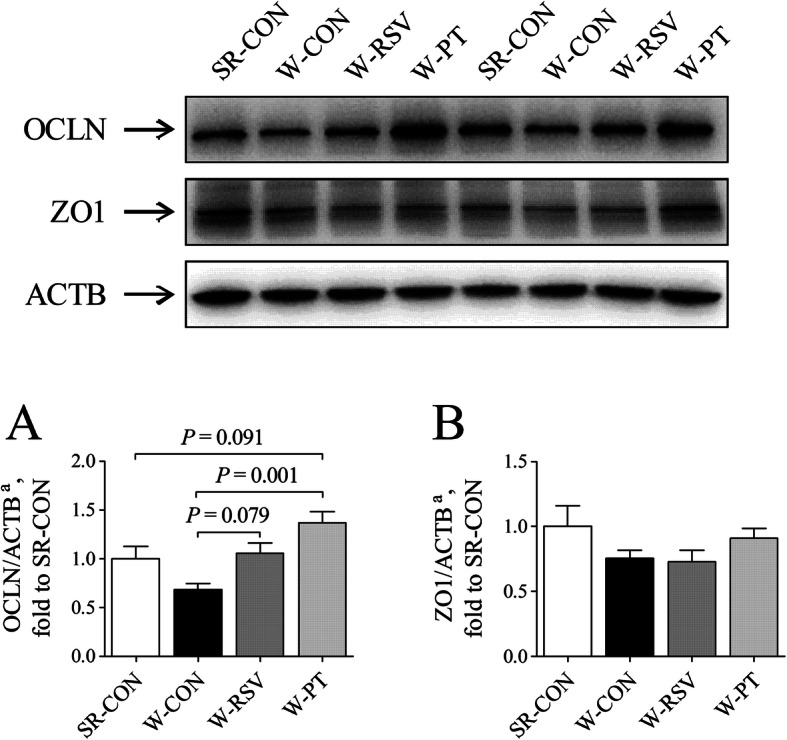
Effects of resveratrol and pterostilbene on protein expression of OCLN (**a**) and ZO1 (**b**) in the jejunum of piglets. The column and its bar represent the mean value and standard error (*n* = 6 piglets/group), respectively. ^a^One-way ANOVA test. *OCLN* occludin, *SR-CON* piglets continued to be nursed by sows aged between 21 and 28 days, *W-CON* piglets were fed a basal diet aged between 21 and 28 days, *W-RSV* piglets were fed a diet supplemented with 300 mg/kg of resveratrol aged between 21 and 28 days, *W-PT* piglets were fed a diet supplemented with 300 mg/kg of pterostilbene aged between 21 and 28 days, *ZO1* tight junction protein 1

A tendency for increased (*P* = 0.079) expression of jejunal OCLN protein (Fig. 5a) was observed in the W-RSV group than in the W-CON group. Similarly, pterostilbene tended to increase the expression of jejunal OCLN protein in the W-PT piglets relative to both the SR-CON (*P* = 0.091) and W-CON (*P* < 0.05) piglets. However, there was no difference in jejunal ZO1 (Fig. 5b) protein content between the groups (*P* > 0.10).

### Jejunal oxidative damage markers

**Fig. 6:**
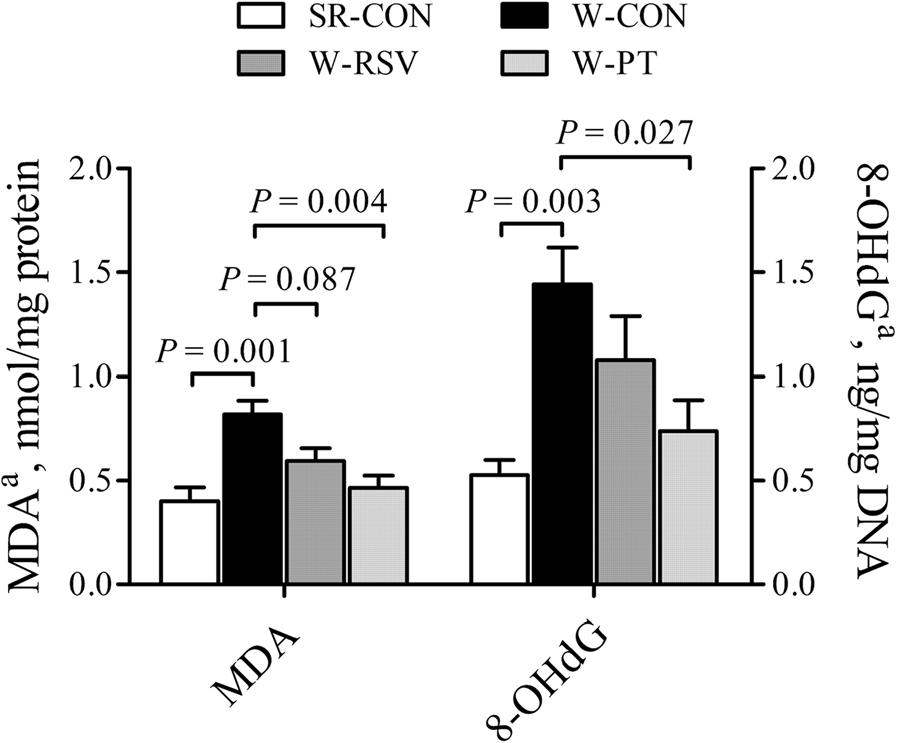
Effects of resveratrol and pterostilbene on the contents of MDA and 8-OHdG in the jejunum of piglets. The column and its bar represent the mean value and standard error (*n* = 6 piglets/group), respectively. ^a^One-way ANOVA test. *MDA* malondialdehyde, *8-OHdG* 8-hydroxy-2´-deoxyguanosine, *SR-CON* piglets continued to be nursed by sows aged between 21 and 28 days, *W-CON* piglets were fed a basal diet aged between 21 and 28 days, *W-RSV* piglets were fed a diet supplemented with 300 mg/kg of resveratrol aged between 21 and 28 days, *W-PT* piglets were fed a diet supplemented with 300 mg/kg of pterostilbene aged between 21 and 28 days

The W-CON group displayed 103.8% and 173.6% higher (*P* < 0.05) jejunal MDA and 8-OHdG contents, respectively, than the SR-CON group (Fig. 6). These increases were significantly alleviated (*P* < 0.05) by pterostilbene but not by resveratrol. A trend toward decreased (*P* = 0.087) MDA content was observed in the W-RSV group, when compared with the W-CON group.

### Jejunal antioxidant capacity

**Fig. 7:**
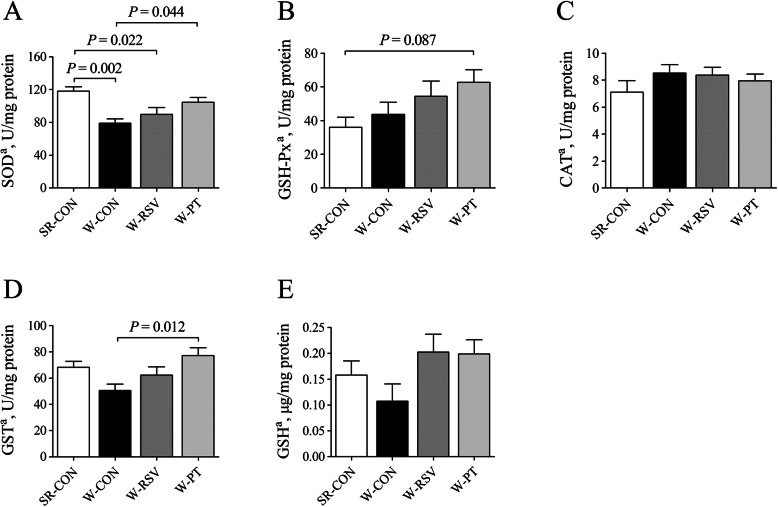
Effects of resveratrol and pterostilbene on SOD (**a**), GSH-Px (**b**), CAT (**c**), and GST (**d**) activities and GSH (**e**) content in the jejunum of piglets. The column and its bar represent the mean value and standard error (*n* = 6 piglets/group), respectively. ^a^One-way ANOVA test. *CAT* catalase, *GSH* reduced glutathione, *GSH-Px* glutathione peroxidase, *GST* glutathione S-transferase, *SR-CON* piglets continued to be nursed by sows aged between 21 and 28 days, *W-CON* piglets were fed a basal diet aged between 21 and 28 days, *W-RSV* piglets were fed a diet supplemented with 300 mg/kg of resveratrol aged between 21 and 28 days, *W-PT* piglets were fed a diet supplemented with 300 mg/kg of pterostilbene aged between 21 and 28 days

The activities of SOD (Fig. 7a) were 33.1% and 24.1% lower (*P* < 0.05) in the jejunal mucosa of W-CON and W-RSV, respectively, compared with the SR-CON piglets. In contrast, in the W-PT group, jejunal SOD activity effectively improved (*P* < 0.05) due to pterostilbene, nearly reaching normal suckling levels. Pterostilbene supplementation also elevated (*P* < 0.05) GST (Fig. 7d) activity in the jejunum of W-PT piglets by 52.7% in comparison with the W-CON piglets. In addition, pterostilbene tended to enhance (*P* = 0.087) jejunal GSH-Px (Fig. 7b) activity in the W-PT piglets when compared with the suckling piglets. No difference between the groups was reflected in CAT (Fig. 7c) activity or GSH (Fig. 7e) content (*P* > 0.10).

### Jejunal mRNA expression of antioxidant enzymes

**Fig. 8:**
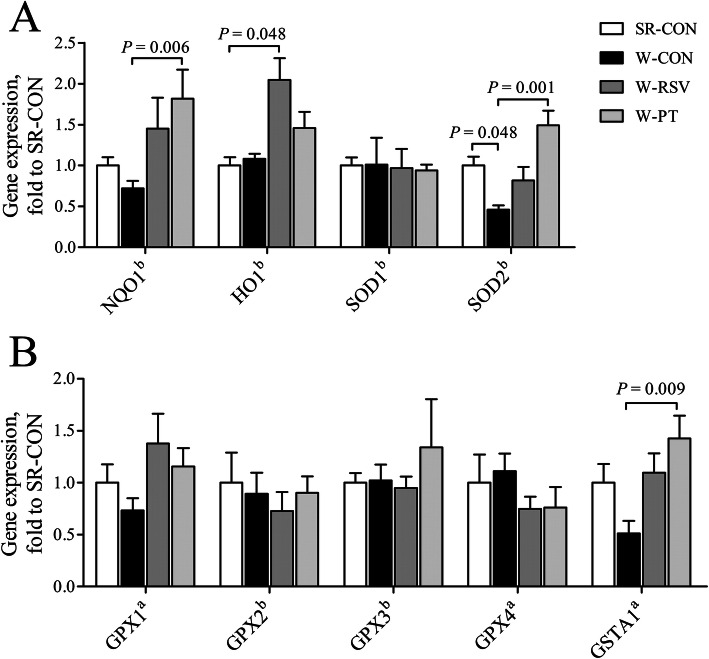
Effects of resveratrol and pterostilbene on antioxidant gene expression (**a** and **b**) in the jejunum of piglets. The column and its bar represent the mean value and standard error (*n* = 6 piglets/group), respectively. ^a^One-way ANOVA test. ^b^Non-parametric Kruskal-Wallis test. *GPX1* glutathione peroxidase 1, *GPX2* glutathione peroxidase 2, *GPX3* glutathione peroxidase 3, *GPX4* glutathione peroxidase 4, *GSTA1* glutathione S-transferase alpha 1, *HO1* heme oxygenase 1, *NQO1* NAD(P)H quinone dehydrogenase 1, *SOD1* superoxide dismutase 1, *SOD2* superoxide dismutase 2, *SR-CON* piglets continued to be nursed by sows aged between 21 and 28 days, *W-CON* piglets were fed a basal diet aged between 21 and 28 days, *W-RSV* piglets were fed a diet supplemented with 300 mg/kg of resveratrol aged between 21 and 28 days, *W-PT* piglets were fed a diet supplemented with 300 mg/kg of pterostilbene aged between 21 and 28 days

Piglets in the W-CON group exhibited a 54.1% decrease (*P* < 0.05) in jejunal *SOD2* (Fig. 8a) mRNA expression relative to that in the SR-CON group. The resveratrol-supplemented diet significantly up-regulated (*P* < 0.05) the mRNA abundance of jejunal *HO1* (Fig. 8a) in the weaned piglets relative to the suckling piglets. In comparison with the W-CON piglets, pterostilbene greatly promoted (*P* < 0.05) the transcriptional expression of *NQO1* (Fig. 8a), *SOD2*, and *GSTA1* (Fig. 8b) in the W-PT piglets by 152.4%, 224.6%, and 179.2%, respectively. No difference between the groups was noted in relation to the expression of *SOD1*, *GPX1*, *GPX2*, *GPX3*, or *GPX4* (*P* > 0.10; Fig. 8a and b).

### Jejunal NRF2 nuclear translocation and downstream target expression

**Fig. 9:**
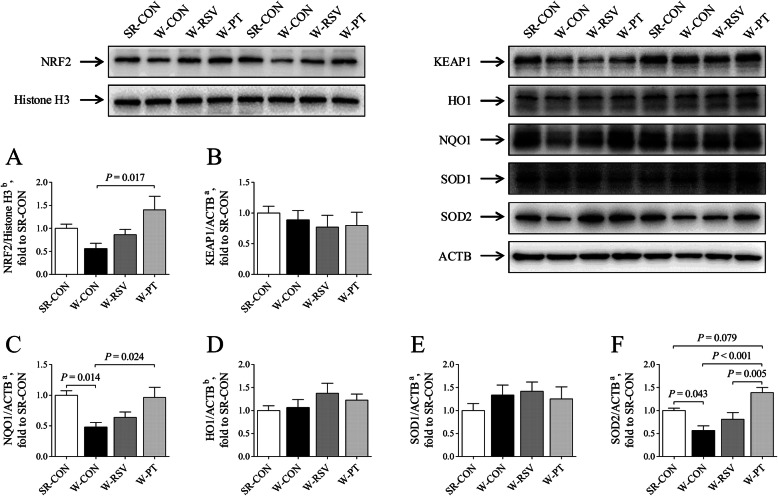
Effects of resveratrol and pterostilbene on NRF2 nuclear translocation (**a**) and the protein expression of KEAP1 (**b**), NQO1 (**c**), HO1 (**d**), SOD1 (**e**), and SOD2 (**f**) in the jejunum of piglets. The column and its bar represent the mean value and standard error (*n* = 6 piglets/group), respectively. ^a^One-way ANOVA test. ^b^Non-parametric Kruskal-Wallis test. *HO1* heme oxygenase 1, *KEAP1* Kelch ECH associating protein 1, *NQO1* NAD(P)H quinone dehydrogenase 1, *NRF2* nuclear factor erythroid 2-related factor 2, *SOD1* superoxide dismutase 1, *SOD2* superoxide dismutase 2, *SR-CON* piglets continued to be nursed by sows aged between 21 and 28 days, *W-CON* piglets were fed a basal diet aged between 21 and 28 days, *W-RSV* piglets were fed a diet supplemented with 300 mg/kg of resveratrol aged between 21 and 28 days, *W-PT* piglets were fed a diet supplemented with 300 mg/kg of pterostilbene aged between 21 and 28 days

Compared with the suckling piglets, weaned piglets fed a basal diet had significantly lower (*P* < 0.05) NQO1 (Fig. 9c) and SOD2 (Fig. 9f) expression levels in the jejunum. In contrast, protein levels of NQO1 and SOD2 had significantly increased (*P* < 0.05) among the W-PT piglets to 101.1% and 144.7%, respectively, compared with those in the W-CON group. Additionally, the SOD2 protein content was greater (*P* < 0.05) in the W-PT group relative to the W-RSV groups. Pterostilbene, as opposed to resveratrol, effectively promoted (*P* < 0.05) the translocation of NRF2 (Fig. 9a) from cytosol into the nucleus by 150.5% in the W-PT piglets, when compared with the W-CON counterparts. There were no evident changes in KEAP1 (Fig. 9b), HO1 (Fig. 9d), and SOD1 (Fig. 9e) protein contents (*P* > 0.10).

## Discussion

The available evidence from rodent and human studies has demonstrated that resveratrol and its derivatives can confer numerous health benefits under certain physiological and pathologic conditions [19, 38, 39]. Likewise, in livestock production, resveratrol has been proven to afford protection against the harmful effects of particularly stressful events, thereby improving animals’ growth performance [10, 13, 14, 40]. In the present study, however, feeding newly-weaned piglets with a resveratrol-supplemented diet exerted no impact on their growth performance during the first week after weaning. These conflicting findings presumably result from variations in resveratrol dosage, the severity of stress, and the growth phase of the animals studied. In particular, the oral absorption of resveratrol may be compromised during the initial period of weaning stress, when the intestinal digestive and absorptive functions are disturbed at different intensities. Supplementation with pterostilbene, but not resveratrol, improved the FE of weanling piglets between the ages of 21 and 28 days; this may be associated with the greater intestinal absorption and enhanced metabolic stability of pterostilbene [18, 24–27]. Consistent with the present observation, pterostilbene was also demonstrated to be more potent than resveratrol in several previous studies [18, 41, 42], indicating that pterostilbene may be a superior candidate for further development.

Early weaning is invariably accompanied by a transient period of intestinal dysfunction as a result of the combined effect of free radicals, inflammatory mediators, in-feed toxins, and other stimulators; this in turn induces a series of typical characteristics of intestinal damage such as villous atrophy, crypt hyperplasia, and an impaired process of epithelial cell turnover [1, 43]. Consistent with the findings in earlier reports [43–45], the W-CON piglets had a lower VH and VH:CD ratio on day 7 post-weaning relative to their sow-reared counterparts in the current study. As the main indicators of the intestinal morphology, the VH and CD directly reflect the absorptive capacity of the mucous membrane [46]. Moreover, in this study, the W-CON piglets exhibited a higher number of villous apoptotic cells but a lower rate of crypt proliferative cells in the jejunum relative to the SR-CON piglets, which may have caused the weaning-induced mucosal damage and thus undermined the integrity of the intestinal barrier [28]. Likewise, it has been demonstrated that early weaning induces apoptosis, enhances cell cycle arrest, and inhibits the expression levels of jejunal genes involving cell proliferation and differentiation in weaned piglets [28, 47].

Generally, it takes approximately 2 weeks for the intestinal architecture of weaned piglets to revert to pre-weaning condition [45], but appropriate nutritional strategies can accelerate recovery from early weaning-associated damage. This is further supported by the results of this study, suggesting that the jejunal VH of weaned piglets partially improved when their basal diet was supplemented with either resveratrol or pterostilbene, which may be associated with the potential of these supplements to mitigate mucosal epithelial damage. This study has observed a beneficial role of pterostilbene in alleviating the excessive apoptosis of jejunal epithelium in weaned piglets; resveratrol also displayed a decreased trend, but not one reaching statistical significance. In addition, resveratrol has been proven to alleviate stress-related damage to small intestines by promoting cell proliferation in mice with hyper-acute ileitis [48] and in broilers under circular heat stress [49]. However, this study has failed to detect the statistically significant alteration of proliferative cell numbers after either resveratrol or pterostilbene supplementation. These inconsistent results may be due to different responses of stress adaptation, which can vary among stimuli and animal species. Therefore, in this study, these stilbenes, particularly pterostilbene, may serve as potent antioxidant or anti-inflammatory modulators and offer direct protection against multiple stimuli harmful to epithelial cell survival. Simultaneously, the administration of pterostilbene reduced plasma DAO activity in W-PT piglets to levels comparable to SR-CON piglets; this is a sensitive indicator of the severity of intestinal damage [50], further confirming the protective role of pterostilbene in attenuating early weaning-associated intestinal damage.

Notably, pterostilbene significantly suppressed the release of *D*-lactate concentration in the plasma of weaned piglets. *D*-lactate is a metabolic end-product of intestinal bacteria that can pass through the epithelial barrier into the blood circulation in cases of intestinal barrier failure [51]. One potential explanation could be the up-regulation of OCLN protein in the jejunum of pterostilbene-fed weaned piglets. The intestinal barrier is physically composed of epithelial cells linked by tight junction complexes, which regulate selective permeability between epithelial cells [52]. Occludin is an important extracellular component of these tight junction complexes and it can limit epithelial permeability to low molecular mass molecules to maintain the barrier function of the small intestine [53]. Notably, the pre-treatment of cultured cells with resveratrol prevented hydrogen peroxide-induced epithelial barrier damage *in vitro* [54]. However, the current data indicate that *in vivo* resveratrol supplementation is less effective than pterostilbene in improving plasma *D*-lactate content and jejunal OCLN expression, mostly because of the differences in the accessibility to the enterocytes, intestinal absorption, and biological potency of each.

The intestine is susceptible to oxidative damage, particularly under stressful circumstances. In pigs, early weaning can induce the overproduction of free radicals and result in a defective antioxidant system in the intestinal tissues and other tissues [4, 5, 47, 55, 56]. This study has observed that the W-CON piglets exhibited diminished jejunal SOD activity relative to the SR-CON piglets, which may be partly due to the down-regulation of SOD2 at both the transcriptional and translational levels. SOD2 encodes manganese SOD, a major antioxidant enzyme residing in the mitochondrial matrix, and breaks down superoxide radicals into hydrogen peroxide, which is further eliminated by either GSH-Px or CAT [57]. Declines in SOD activity caused by weaning stress have been demonstrated in several studies, including one by Zhu et al. [4]. In addition, early weaning reduced the NQO1 protein content in the jejunum of weaned piglets. NQO1 is a flavoenzyme that catalyzes a two-electron reductive metabolism and detoxifies quinones and their derivatives, thereby protecting cells against redox cycling and oxidative stress [58]. Such defects in SOD2 and NQO1 expression, combined with inferior SOD activity, may account for the increased contents of MDA and 8-OHdG (the biomarkers of oxidative stress to lipids and DNA, respectively); collectively, these data support the possibility that weaning stress results in a collapse in the jejunal redox system and clearly induces significant oxidative damage among young piglets.

After treatment with pterostilbene, the MDA and 8-OHdG concentrations were reduced and nearly identical to normal suckling levels, while only a tendency for decreased MDA content was observed following resveratrol supplementation. The attenuated oxidative damage may be due to the ability of stilbenes to scavenge free radicals, such as hydrogen peroxide and superoxide anion, as they contain hydroxyl groups [38]. Structure-activity relationship analysis has revealed that the 4′ hydroxyl group is the most reactive site as a result of the resonance effects occurring between the two aromatic rings; therefore, it plays a dominant role in the free-radical scavenging activity of stilbenes [59–61]. As demonstrated in previous research on the redox reactions of resveratrol and its derivatives, pterostilbene, which has a free hydroxyl group in the 4′ position, exhibits comparable or superior antioxidant activity relative to its parent compound [59, 62]. Likewise, Mikstacka et al. [63] have reported that pterostilbene is more effective in scavenging lipid peroxyl radicals than resveratrol due to its higher lipophicity. Therefore, the results may provide a potential explanation for the antioxidant action of pterostilbene in reducing jejunal DNA oxidative damage and lipid peroxidation in weaned piglets.

Aside from its antioxidant activity, pterostilbene also produced an enhancement of antioxidant enzymes in the jejunum of weaned piglets, as made evident by higher SOD and GST activities and the partially increased level of GSH-Px activity. Importantly, pterostilbene was found to increase the mRNA levels of jejunal *NQO1*, *SOD2*, and *GSTA1* and to promote the expression of NQO1 and SOD2 at the translational level. This occurs due to the increased accumulation of nuclear NRF2 protein stimulated by pterostilbene. NRF2 activation and its translocation from cytosol into the nucleus facilitates NRF2’s binding to antioxidant response elements and the transcriptional expression of its downstream targets, such as HO1, NQO1, and SOD2 [64]. Pterostilbene has recently been identified as a potent NRF2 activator in both *in vitro* and *in vivo* experiments [41, 65–67]; it interacts directly with the basic amino acids of the kelch domain of KEAP1 and induces a dissociation of the KEAP1-NRF2 complex, subsequently facilitating the nuclear translocation of NRF2 [65]. This suggests that feeding weaned piglets a pterostilbene-supplemented diet could restore the activities of NRF2’s downstream antioxidant enzymes in the W-PT piglets, by comparison with the W-CON counterparts; this could therefore prevent the accumulation of DNA oxidative damage and lipid peroxidation products in the jejunum. These pterostilbene benefits may therefore work together to prevent or mitigate the intestinal oxidative damage arising from early weaning.

## Conclusion

This study provided the first detailed evidence that supplementation with either resveratrol or pterostilbene may have the potential to alleviate early weaning-induced damage to villus morphology and epithelial cell survivals in the jejunum of young piglets. Notably, pterostilbene is more effective than its parent compound in mitigating jejunal oxidative stress, most likely as a result of its ability to facilitate NRF2 signals and crucial antioxidant enzyme activities, which consequently preserves the permeability of the small intestine and improves the FE of weanling piglets. The data presented herein indicate that pterostilbene could be a promising anti-stress supplement for newly-weaned piglets.

## Data Availability

The datasets produced and/or analyzed during the current study are available from the corresponding author on reasonable request.

## Abbreviations

ACTB: Beta actin
ADFI: Average daily feed intake
ADG: Average daily gain
ANOVA: One-way analysis of variance
CAT: Catalase
CD: Crypt depth
cDNA: Complementary DNA
DAO: Diamine oxidase
DAPI: 4′-6-diamidino-2-phenylindole
FE: Feed efficiency
GAPDH: Glyceraldehyde phosphate dehydrogenase
GPX1: Glutathione peroxidase 1
GPX2: Glutathione peroxidase 2
GPX3: Glutathione peroxidase 3
GPX4: Glutathione peroxidase 4
GSH: Reduced glutathione
GSH-Px: Glutathione peroxidase
GST: Glutathione S-transferase
GSTA1: Glutathione S-transferase alpha 1
HO1: Heme oxygenase 1
KEAP1: Kelch ECH associating protein 1
MDA: Malondialdehyde
NQO1: NAD(P)H quinone dehydrogenase 1
NRF2: Nuclear factor erythroid 2-related factor 2
OCLN: Occludin
8-OHdG: 8-hydroxy-2´-deoxyguanosine
PBS: Phosphate buffer solution
PCR: Polymerase chain reaction
SE: Standard error
SOD: Superoxide dismutase
SOD1: Superoxide dismutase 1
SOD2: Superoxide dismutase 2
SPSS: Statistical Package for the Social Sciences
TBST: Tris-buffered saline plus 0.2% Tween-20
TUNEL: Terminal deoxynucleotidyl transferase deoxyuridine triphosphate nick end labeling
VH: Villus height
ZO1: Tight junction protein 1
